# Psychological Profiles, Stress, Sleep Quality, and Psychological Risk Indicators Across Teams and Competitive Levels in Elite Football Players

**DOI:** 10.3390/bs16101738

**Published:** 2026-09-24

**Authors:** Eduardo Morelló, Jon Larruskain, Laura Gil-Caselles, Aurelio Olmedilla-Zafra

**Affiliations:** 1 Department of Sport Performance, Athletic Club de Bilbao, 48009 Bilbao, Spain; e.morellotomas@um.es (E.M.); j.larruskain@athletic-club.eus (J.L.); 2 Research Group HUMSE, Faculty of Sports Sciences, University of Murcia, 30720 Murcia, Spain; 3 Research Group HUMSE, Department of Personality, Evaluation and Psychological Treatment, Faculty of Psychology, University of Murcia, 30100 Murcia, Spain; olmedilla@um.es

**Keywords:** stress, anxiety, depressive symptoms, sleep quality, psychological risk indicators, elite football, team comparisons, psychological monitoring

## Abstract

Psychological factors and sleep quality are relevant to athlete well-being and adaptation in high-performance sport, yet their distribution within the same elite football structure remains insufficiently understood. This pooled cross-sectional study described and compared psychological profiles and psychological risk indicators across 11 teams within a single elite football organisation and explored selected contextual variables. Participants were 376 elite football players (245 male and 131 female; aged 14–37 years) from teams competing at U16, U19, amateur, and professional levels. Assessment followed the Psycholight framework and incorporated selected dimensions from the CPRD, MIPS, STAI, DASS-21, and PSQI. Group differences were examined using nonparametric comparisons, Wilcoxon effect sizes, Fisher’s exact tests, and odds ratios with 95% confidence intervals. Psychological profiles and risk distributions varied across the studied teams, with heterogeneous patterns across psychological and sleep-related indicators. Given the close interrelationship between sex, age, team, competitive level, and season, these findings should be interpreted as patterns observed among the teams represented in the present sample rather than as independent effects of sex or competitive level.

## 1. Introduction

In recent years, psychological well-being and mental health in high-performance sport have received increasing attention in both research and professional practice, particularly because of the need to identify and address signs of psychological distress in elite athletes at an early stage (Gulliver et al., 2012; Reardon et al., 2019). In highly demanding environments, variables such as stress, anxiety, mood, perfectionism, motivation, and stress control may influence how athletes respond to training, competition, and broader social demands. These variables have been associated with athletes’ psychological adaptation, well-being, and responses to the demands of training and competition, as well as with vulnerability to adverse outcomes, including impaired performance and injury. However, these relationships should be understood within a multifactorial framework rather than interpreted as direct or causal associations (Gil-Caselles et al., 2025; Ivarsson et al., 2017; Johnson & Ivarsson, 2025; Sánchez-Ruiz et al., 2025; Tranaeus et al., 2024).

This issue is particularly relevant in elite football, where physical, competitive, and social demands extend from developmental stages through to the professional level. Selection and deselection processes, transitions between age groups and competitive categories, performance expectations, and uncertainty associated with athletic progression may shape players’ psychological experiences (Mills et al., 2012; Saward et al., 2020; Swainston et al., 2020). In this context, systematic assessment of psychological functioning may help identify players with differing support needs and inform monitoring, referral, and intervention strategies (Gouttebarge et al., 2021). Importantly, psychological indicators should be interpreted as signs of potential distress or less adaptive functioning rather than as clinical diagnoses or direct predictors of injury or impaired performance (Johnson & Ivarsson, 2025; Purcell et al., 2019; Reardon et al., 2019).

Psychological functioning in sport is inherently multidimensional. Performance-related characteristics such as motivation, stress control, concentration, self-confidence, mental preparation, and coping have been associated with athletes’ adaptation, engagement, expertise development, and competitive performance, although these relationships depend on the athlete, the task, and the sporting context (Mahoney et al., 1987; Mahoney & Meyers, 2021; Fawver et al., 2020; Lochbaum et al., 2022). The CPRD provides a multidimensional assessment of several of these characteristics and has been used to characterize psychological profiles across different sports and competitive contexts (Gimeno et al., 2001; Olmedilla et al., 2018, 2019; Saward et al., 2023).

Other psychological and health-related domains provide complementary information. Maladaptive perfectionism has been associated with competitive anxiety, fear of failure, psychological distress, and burnout, whereas more adaptive perfectionistic tendencies may be associated with favorable motivational and performance-related outcomes (Stoeber, 2011; Stoeber et al., 2006; Hill et al., 2018). Trait anxiety reflects a relatively stable tendency to perceive situations as threatening, while anxiety responses in sport are also shaped by situational appraisal, coping resources, interpersonal conditions, and competitive demands (Spielberger et al., 1983; Mahoney & Meyers, 2021; Hanin, 2021; Hackfort & Spielberger, 2021; Horikawa & Yagi, 2012). Symptoms of depression, anxiety, and stress provide additional information about emotional functioning and may reflect psychological distress requiring closer monitoring, although they should not be equated with psychiatric diagnoses (Purcell et al., 2019; Reardon et al., 2019; Vaughan et al., 2020). Sleep quality is likewise relevant to recovery, emotional regulation, cognitive functioning, and readiness to train and compete, and may be affected by training schedules, competition, travel, pre-competition arousal, and personal or academic demands (Fullagar et al., 2015; Walsh et al., 2021; Gupta et al., 2017).

Although these domains represent heterogeneous and partly overlapping constructs, their joint assessment may provide a descriptive summary of different dimensions of psychological functioning. In the present framework, the cumulative psychological risk index is not assumed to represent a single latent psychological construct. Rather, it is used exclusively as an exploratory descriptive composite score integrating information across performance-related characteristics, maladaptive psychological tendencies, emotional symptoms, and sleep quality, while retaining the individual component scores for interpretation. Equal weighting of the components represents a parsimonious operational approach rather than an assumption that all domains have identical theoretical or clinical importance. Accordingly, the composite score should be interpreted cautiously and descriptively, alongside its individual components and the athlete’s developmental, competitive, and personal context. It should not be interpreted as a validated screening, diagnostic, prognostic, or decision-making measure. Such an integrated approach may provide a structured way of organizing multidimensional psychological information for descriptive purposes and further evaluation (Gouttebarge et al., 2021; Purcell et al., 2019; Reardon et al., 2019).

Despite the relevance of these variables, studies examining psychological functioning, emotional distress, sleep quality, and psychological risk indicators in an integrated manner across multiple teams within the same elite football organization remain limited. Examining teams embedded within a shared organizational structure may provide a complementary perspective to studies conducted across different clubs or sporting contexts, as it allows psychological variation to be described across teams while part of the broader organizational environment and monitoring framework is shared. Although this approach does not eliminate important differences between teams, including age, competitive demands, and other team-specific characteristics, it may help characterize within-organization patterns in psychological functioning and sleep. From an applied perspective, such information may provide a descriptive basis for future research and for the contextual interpretation of psychological assessments across different stages of the developmental pathway from youth to professional football.

Accordingly, the primary aim of the present study was to describe and compare psychological profiles and psychological risk indicators across different teams within the same elite football organization. Rather than estimating independent effects of sex or competitive level, the study examined patterns across teams that differed simultaneously in several characteristics, including sex composition, age, competitive level, and season. In addition, exploratory analyses examined whether psychological risk indicators varied according to competitive participation, playing position, living arrangements, years at the club, and body-composition status, operationalized through team-specific tertiles of the sum of six skinfolds. These contextual analyses were intended to identify potential subgroup patterns that could inform future hypothesis-driven research rather than to test established theoretical predictions.

Given the descriptive and context-dependent nature of these comparisons, we expected psychological profiles and psychological risk distributions to vary across the teams represented in the academy, without assuming that such differences reflected independent effects of sex or competitive level or followed a uniform linear gradient across competitive stages. Analyses involving competitive participation, playing position, living arrangements, years at the club, and body-composition status were considered exploratory, and no directional hypotheses were established.

## 2. Materials and Methods

### 2.1. Design

This study used an observational, descriptive, pooled cross-sectional design based on baseline psychological assessments collected across the 2023/24 and 2024/25 competitive seasons. The analyses compared psychological profiles, sleep quality, and psychological risk indicators across predefined competitive and developmental categories within a single elite football academy. Reporting followed the Strengthening the Reporting of Observational Studies in Epidemiology (STROBE) guidelines for cross-sectional studies (von Elm et al., 2007).

### 2.2. Participants

The study included 376 unique elite football players from a single high-performance football academy, aged 14 to 37 years (M = 20.3, SD = 5.3), including 245 male and 131 female players. Data were obtained during the 2023/24 and 2024/25 competitive seasons. Because players could contribute a baseline assessment in each season in which they were included, the analytical dataset comprised 500 baseline player-season observations. Each player contributed a maximum of one baseline psychological assessment per competitive season. Consequently, some players contributed observations in both seasons, whereas others contributed an observation in only one season. The team-specific sample sizes reported below therefore refer to player-season observations and should not be interpreted as mutually exclusive numbers of unique players.

For analysis, the club teams were organized into eight competitive/developmental categories: men’s first team (M1; *n* = 41), men’s second/third teams (M2/3; *n* = 94), men’s U19 group (*n* = 105), men’s U16 group (*n* = 98), women’s first team (F1; *n* = 32), women’s second team (F2; *n* = 39), women’s third team (F3; *n* = 56), and women’s U16 group (U16F; *n* = 35). The men’s first team competed in Spanish LaLiga; the men’s second and third teams competed in the Spanish third and fourth divisions, respectively; and the women’s first, second, and third teams competed in the Spanish first, third, and fourth divisions, respectively. The developmental categories were organized according to the club’s age-structured pathway: the U16 analytical group comprised the U15–U16 developmental stage, whereas the U19 analytical group comprised the U17–U19 developmental stage. The men’s second and third teams were pooled as a senior sub-elite category immediately below the first-team level. These groupings were based on the club’s competitive and developmental structure and were intended to represent comparable stages of the football pathway.

Because the developmental categories were inherently age-structured, age and competitive category were closely linked within these groups. Accordingly, the overall observed age characteristics of the sample are reported above, while age was not treated as an independent grouping variable in the team comparisons. For the purposes of this study, the variable labelled sex corresponded to the male or female competition category in which players were registered within the club structure; self-identified gender was not assessed as a study variable.

### 2.3. Measures

Psychological assessment was conducted using the measures included in the Psycholight protocol (Olmedilla-Zafra & García-Mas, 2023). The same assessment framework was used in Morelló et al. (2026), although the analytical purpose of that study differed from that of the present manuscript, as detailed in the Procedure section. Psycholight is a systematic framework designed to assess psychological functioning and potential indicators of psychological vulnerability in applied high-performance sport settings. For the present study, nine psychological and sleep-related components were considered: motivation, stress control, influence of performance evaluation, maladaptive perfectionism, trait anxiety, symptoms of depression, anxiety and stress, and global sleep quality. All instruments were administered in validated Spanish-language versions. Reliability estimates reported in previous validation studies are provided below as additional information regarding the psychometric properties of the measures. Scoring followed the corresponding published procedures and the interpretive criteria incorporated into the Psycholight protocol.

#### 2.3.1. Psychological Characteristics Related to Sport Performance Questionnaire

Psychological characteristics related to sport performance were assessed using the *Cuestionario de Características Psicológicas Relacionadas con el Rendimiento Deportivo* (Macías; Gimeno et al., 2001). The CPRD comprises 55 items distributed across five subscales: stress control, influence of performance evaluation, motivation, mental skills, and team cohesion. Items are rated on a five-point response scale. In the present study, three of the five CPRD subscales were included: motivation, stress control, and influence of performance evaluation. These subscales were selected because they form part of the Psycholight psychological risk framework. Higher scores indicate more favorable psychological characteristics related to sport performance. Previous validation studies have reported acceptable internal consistency for the CPRD (Gimeno et al., 2001).

#### 2.3.2. Multidimensional Inventory of Perfectionism in Sport

Sport-specific perfectionism was assessed using the Multidimensional Inventory of Perfectionism in Sport (MIPS; Stoeber et al., 2006; Atienza et al., 2020). The instrument assesses two broad dimensions: perfectionistic strivings and negative reactions to imperfection. In the present study, one of the two MIPS dimensions was included: negative reactions to imperfection, which was used as an indicator of maladaptive perfectionism within the psychological risk framework. Higher scores reflect greater concern about mistakes and more negative responses to perceived failure or imperfection. Previous validation studies of the Spanish version have reported adequate internal consistency for the dimensions of the instrument (Atienza et al., 2020).

#### 2.3.3. State–Trait Anxiety Inventory

Trait anxiety was assessed using the State–Trait Anxiety Inventory (STAI; Spielberger et al., 1983; Buela-Casal et al., 2015). The STAI comprises separate state and trait anxiety subscales. In the present study, only the trait anxiety subscale was included. This subscale comprises 20 items and assesses relatively stable individual differences in the tendency to perceive situations as threatening and to respond with heightened anxiety. Items are rated on a four-point response scale, with higher scores indicating greater trait anxiety. The Spanish version has shown adequate internal consistency for the trait anxiety subscale (approximately α = 0.80; Buela-Casal et al., 2015).

#### 2.3.4. Depression Anxiety Stress Scales–21

Symptoms of depression, anxiety, and stress were assessed using the Depression Anxiety Stress Scales–21 (DASS-21; Lovibond & Lovibond, 1995; Bados et al., 2005). The instrument comprises 21 items distributed across three seven-item subscales: depression, anxiety, and stress. All three DASS-21 subscales were included in the present study. Participants indicated the extent to which each statement had applied to them during the previous week using a four-point response scale ranging from 0 (*did not apply to me at all*) to 3 (*applied to me very much or most of the time*). Higher scores indicate greater symptom severity. Adequate internal consistency has also been reported for the Spanish DASS-21, with Cronbach’s alpha coefficients of 0.84 for depression, 0.70 for anxiety, and 0.82 for stress (Bados et al., 2005).

#### 2.3.5. Pittsburgh Sleep Quality Index

Sleep quality was assessed using the Pittsburgh Sleep Quality Index (PSQI; Buysse et al., 1989; Royuela Rico & Macías Fernández, 1997). The PSQI comprises 19 self-report items grouped into seven components: subjective sleep quality, sleep latency, sleep duration, habitual sleep efficiency, sleep disturbances, use of sleep medication, and daytime dysfunction. These components are combined to produce a global score ranging from 0 to 21. In the present study, only the global PSQI score was included, and the seven component scores were not analyzed separately. Higher global scores indicate poorer sleep quality. For the Spanish version of the PSQI, adequate internal consistency has been reported for the global score (α = 0.81; Royuela Rico & Macías Fernández, 1997).

Together, the selected subscales, dimensions, and global scores provided a multidimensional assessment of psychological functioning, emotional distress, and sleep quality. They were used to characterize players’ psychological profiles and to construct the global psychological risk index.

### 2.4. Procedure

Data were collected during the 2023/24 and 2024/25 competitive seasons. For the present study, a maximum of one baseline psychological assessment per player and competitive season, conducted at the beginning of the corresponding season, was included in the analyses. Participants completed the selected measures included in the Psycholight protocol in digital format within the club’s usual training environment. Assessments were administered and supervised by members of the club’s psychology staff in accordance with the standardized procedures established for the protocol (Olmedilla-Zafra & García-Mas, 2023). Because these data were derived from the club’s routine athlete-monitoring programme rather than from a prospectively recruited research cohort, a conventional eligible–excluded participant flow was not recorded in the same manner as in a recruitment-based study.

The psychological assessments were conducted by Athletic Club as part of its routine athlete-monitoring programme and were not initiated specifically for the purposes of the present research. No additional assessments were administered for this study. At the beginning of the corresponding season, adult players and, where applicable, parents or legal guardians of underage players authorized participation in the club’s routine medical and psychological assessments. Underage players also provided assent.

The original monitoring data remained under the custody of the Athletic Club and were managed by the club’s psychology professionals in accordance with its internal confidentiality, ethical, and data-protection procedures. For the purposes of the present study, the data were handled confidentially and anonymized before research analysis. The present study therefore constituted a secondary analysis of data originally collected as part of Athletic Club’s routine psychological monitoring programme. Athletic Club formally authorized the use of these anonymized data for the present study. The secondary analysis was conducted in accordance with the ethical principles of the Declaration of Helsinki (World Medical Association, 2025).

Relationship with the previous Psycholight study. The baseline psychological assessments collected during the 2023/24 season and included in the present dataset overlap with those previously analysed by Morelló et al. (2026). Specifically, Morelló et al. (2026) included 281 players from the 2023/24 season and examined the prospective association between the Psycholight composite psychological score and subsequent sports injury incidence. The present study extends the dataset by incorporating baseline assessments from the 2024/25 season and addresses a different research question. No injury outcomes are analysed here; instead, the present analyses describe and compare psychological profiles, sleep quality, individual psychological indicators, and the exploratory composite score across competitive and developmental team categories, together with exploratory analyses of selected contextual variables. Accordingly, although the two studies share the 2023/24 baseline psychological data and the Psycholight assessment framework, the 2024/25 observations and the team-category and contextual analyses reported in the present manuscript are specific to this study.

### 2.5. Psychological Risk Index

A cumulative psychological risk index was constructed according to the criteria of the Psycholight protocol (Olmedilla-Zafra & García-Mas, 2023) and the operationalization previously applied by Morelló et al. (2026). The index comprised nine components: stress control, influence of performance evaluation, and motivation, assessed using the CPRD; maladaptive perfectionism, assessed using the MIPS; trait anxiety, assessed using the STAI; symptoms of depression, anxiety, and stress, assessed using the DASS-21; and global sleep quality, assessed using the PSQI.

Each component was classified into three risk levels, low, medium, and high, according to the interpretation criteria and cut-off scores established for each instrument and integrated into the Psycholight protocol. These levels were coded as 0, 1, and 2, respectively. The sum of the nine components yielded a raw score ranging from 0 to 18, with higher scores indicating greater cumulative psychological vulnerability. This score was subsequently rescaled to a 0–10 metric to facilitate interpretation using the following formula: Rescaled score = raw score × 10/18.

Based on the cumulative score, participants were classified into three global psychological risk categories. Raw scores from 0 to 4 were classified as low risk, scores from 5 to 9 as medium risk, and scores of 10 or above as high risk. For interpretability, the raw score was linearly rescaled to a 0–10 metric; however, classification into low-, medium-, and high-risk categories was based exclusively on the raw-score thresholds described above.

The composite score was used exclusively as an exploratory descriptive summary of the nine included components. It has not been validated as an independent psychometric construct or as a screening, diagnostic, prognostic, or decision-making tool. No assumption is made that the composite score provides incremental information beyond that contained in its individual components, including established measures such as the DASS-21 and PSQI. Accordingly, its results should be interpreted descriptively and alongside the individual component scores.

### 2.6. Statistical Analysis

Data preprocessing and statistical analyses were conducted using R, version 4.4.1. The unit of analysis was the baseline player-season observation. Because some players contributed one baseline assessment in each of the two competitive seasons, repeated observations were present within the dataset. The original analytical approach did not explicitly model within-player dependence across seasons or the hierarchical clustering of players within teams. Accordingly, the analyses were used to describe and compare patterns across the specific analytical team categories rather than to estimate independent effects of sex, age, competitive level, team, or season. The associated *p*-values, odds ratios, and confidence intervals should therefore be interpreted as exploratory inferential summaries accompanying these descriptive comparisons, rather than as estimates of independent effects. Psychological and sleep-related variables were summarized using the median and interquartile range, expressed as the 25th and 75th percentiles.

Pairwise comparisons were conducted between teams and, within each sex, between groups defined according to the contextual variables considered: playing position, years at the club, sum of six skinfolds, competitive participation, and living arrangements. Comparisons were performed using the Mann–Whitney *U* test. Effect sizes were estimated using Wilcoxon’s *r* and interpreted as small, moderate, and large using reference values of 0.10, 0.30, and 0.50, respectively. Team comparisons constituted the primary analyses, whereas analyses involving playing position, years at the club, sum of six skinfolds, competitive participation, and living arrangements were considered exploratory.

For each psychological variable and for the global index, the proportions of participants classified as low, medium, and high risk were calculated. For inferential analyses, participants classified as medium or high risk were compared with those classified as low risk. Group differences were examined using Fisher’s exact test, and odds ratios (ORs) with 95% confidence intervals were calculated.

To account for multiple pairwise comparisons, *p*-values for comparisons across the levels of each grouping factor were adjusted separately for each psychological variable using the Benjamini–Hochberg false discovery rate procedure. Statistical significance was set at *p* < 0.05.

## 3. Results

### 3.1. Differences Across Teams

Descriptive analyses showed heterogeneous psychological profiles across the teams included in the study. Differences between teams were observed across performance-related characteristics, emotional distress indicators, sleep quality, and the psychological risk index. Given the close interrelationship between sex, age, team, competitive level, and season, these patterns should not be interpreted as independent effects of sex or competitive level. Detailed team-level results and pairwise comparisons are presented in Table 1.

### 3.2. Distribution of Psychological Risk

Risk distributions also varied across the teams included in the study, with heterogeneous proportions of players classified as low, medium, or high risk across psychological and sleep-related variables. These differences were interpreted as team-specific descriptive patterns rather than as independent effects of sex or competitive level. Differences in the proportion of players classified as medium or high risk were quantified using odds ratios, as shown in Table 2.

### 3.3. Distribution of Psychological Risk According to Contextual Variables

Exploratory analyses of contextual variables identified several subgroup differences in the distribution of psychological risk indicators. These findings varied across contextual factors and teams and should not be interpreted as independent associations because age, team, competitive level, season, and other relevant characteristics were not simultaneously adjusted for. The complete distributions and between-group comparisons are presented in Table 3 and Table 4.

## 4. Discussion

Psychological profiles, sleep quality, and psychological risk distributions varied across the teams included in this elite football academy. The observed patterns were heterogeneous and did not show a consistent linear gradient across competitive stages. Descriptive differences were also observed between male and female teams; however, because sex, age, team, competitive level, and season are closely intertwined in the present sample, these findings cannot be interpreted as independent effects of sex or competitive level.

Previous research in football has reported differences between male and female players in symptoms of depression and anxiety, although such patterns may vary according to age, competitive group, sporting status, and other contextual factors (Junge & Feddermann-Demont, 2016; Junge & Prinz, 2019; Perry et al., 2022). More broadly, psychological differences between males and females show substantial overlap and may depend on contextual and measurement conditions (Hyde, 2014). Organizational and psychosocial conditions may also differ across sporting environments (Pascoe et al., 2022). Accordingly, the present findings should be interpreted as patterns observed within this particular academy rather than as inherent characteristics of male or female football players.

Some lower-level teams, particularly the female U16 team, showed less favorable profiles across several indicators. However, competitive level cannot be isolated from age, developmental stage, team, and other characteristics in the present design. Previous research has highlighted the psychological demands associated with developmental transitions, selection processes, and progression through elite football pathways (Mills et al., 2012; Saward et al., 2020; Swainston et al., 2020), while studies of talented young players have emphasized the heterogeneity of mental-health profiles across developmental contexts (Tachom Waffo & Hauw, 2024). Research comparing competitive levels has likewise yielded heterogeneous findings (Pettersen et al., 2022; Ruiz-Esteban et al., 2020). These findings therefore support a team- and context-specific interpretation rather than a simple linear relationship between competitive level and psychological functioning.

The analysis of psychological risk categories complemented the comparison of continuous scores by describing how less favorable indicators were distributed across teams. Previous reviews have shown that anxiety, depression, psychological distress, and sleep disturbances are relatively common among elite athletes, although prevalence estimates vary considerably depending on instruments, thresholds, sports, and populations (Gouttebarge et al., 2019; Rice et al., 2016). Morelló et al. (2026) previously applied the Psycholight framework to summarize several psychological dimensions in football players. Nevertheless, the composite index used here should be regarded as an exploratory descriptive operational summary of heterogeneous psychological and sleep-related indicators rather than as a validated diagnostic or predictive construct. Its interpretation should therefore remain subordinate to the individual component scores and the specific context of the athlete.

Sleep quality also differed across teams and contributed to the cumulative index. Sleep is relevant to emotional regulation, cognitive functioning, recovery, and adaptation to training and competition (Walsh et al., 2021), and poorer sleep has been associated with symptoms of depression, anxiety, and stress in elite athletes (Facer-Childs et al., 2022). However, the cross-sectional design does not allow the direction of these relationships to be established.

Exploratory analyses also identified differences according to competitive participation, playing position, living arrangements, years at the club, and body-composition status. These findings should not be interpreted as independent correlates of psychological risk because these variables were not simultaneously adjusted for age, team, competitive level, and season. Previous research suggests that match involvement may be related to psychological well-being over time (Madsen et al., 2025), and social support has been associated with mental-health outcomes in athletes (Delfin et al., 2024), but the present study did not directly assess the mechanisms underlying the observed subgroup differences. Explanations involving stigma, emotional disclosure, family support, autonomy, selection processes, or organizational demands therefore remain hypotheses for future research rather than explanations supported by the present data (Gulliver et al., 2012; Rice et al., 2016).

### 4.1. Practical Implications

From an applied perspective, systematic multidimensional psychological assessment may provide a structured way of organizing information about psychological functioning and sleep within elite football settings (Gouttebarge et al., 2021; Olmedilla-Zafra & García-Mas, 2023). However, the present study does not establish that the composite psychological risk index can identify athletes who require intervention, predict subsequent outcomes, or guide referral decisions. The index should therefore be interpreted cautiously and alongside its individual components, the athlete’s circumstances, and professional assessment rather than as a diagnostic or decision-making instrument.

Evidence that psychological interventions may reduce performance anxiety in athletes (Niering et al., 2023) highlights the broader relevance of psychological support in sport, but future prospective research is required to determine whether the indicators examined here have practical value for identifying support needs or informing intervention decisions.

### 4.2. Limitations

Several limitations should be considered. First, the pooled cross-sectional design precludes conclusions regarding temporal sequence or causality. In addition, sex, age, team, competitive level, and season were closely interrelated in this sample, resulting in substantial confounding and limiting the interpretation of differences as independent effects of any one characteristic. Players were also clustered within teams, whereas the primary analyses did not explicitly model this hierarchical structure.

The dataset additionally included repeated observations across seasons. Although the study comprised 376 unique players, the analyses included 500 baseline player-season observations because some players contributed a baseline assessment in both competitive seasons. Within-player dependence across seasons was not explicitly modelled. Accordingly, the inferential statistics reported in the present study should be interpreted cautiously and primarily as exploratory indicators of the observed group patterns. These features should be considered when interpreting the between-team comparisons, which are intended to describe patterns within the specific team categories studied rather than independent effects of age, sex, competitive level, team, or season. Future studies should use multilevel, repeated-measures, or related analytical approaches capable of accounting simultaneously for repeated observations within players and clustering within teams.

In addition, the large number of team and subgroup comparisons increases the risk of chance findings despite application of the Benjamini–Hochberg correction, and isolated significant results should therefore be interpreted cautiously. The categorization of continuous variables such as competitive participation and skinfold measures into tertiles may also have resulted in some loss of information and sensitivity to the selected thresholds; these analyses should therefore be regarded as exploratory.

All measures were based on self-report and may have been influenced by recall, response interpretation, perceived confidentiality, and social desirability (van de Mortel, 2008). Measurement invariance across sex, age groups, competitive levels, or teams was not examined, and it therefore cannot be assumed that the instruments functioned equivalently across all subgroups. Reliability coefficients were not estimated specifically in the present sample; therefore, the psychometric performance of the measures in this particular population requires further examination. Similarly, the applicability of the risk thresholds across the broad age range represented in the sample remains uncertain.

The composite psychological risk score also requires further validation. It combines heterogeneous and partly overlapping constructs using equal weights and global classification thresholds. Reliability of the composite score, correlations among its components, sensitivity analyses of alternative continuous formulations, and incremental information beyond established measures such as the DASS-21 and PSQI were not evaluated in the present study. Its construct validity, classification accuracy, sensitivity to change, and prospective utility therefore remain undetermined. Consequently, the score should be interpreted exclusively as an exploratory descriptive operational summary of the included components and not as a validated measure of psychological risk, screening instrument, or decision-making tool.

Finally, all participants belonged to a single elite football academy, limiting generalizability to other clubs, competitions, countries, and sporting structures. Several potentially relevant variables, including injury status, training and competition load, academic demands, contract status, perceived coach support, family support, previous mental-health history, and access to psychological services, were not measured and could not be considered as potential explanatory factors.

### 4.3. Future Research

Future studies should use longitudinal and multilevel or repeated-measures designs capable of distinguishing variability attributable to individual players, repeated assessments, teams, developmental stages, competitive levels, seasons, and organizational environments. Research involving multiple academies, clubs, competitions, and countries would help determine whether the patterns observed here can be replicated across different football systems.

Future work should also directly assess contextual mechanisms that could not be examined in the present study, including perceived coach support, social and family support, autonomy, satisfaction with living arrangements, academic or occupational demands, contract security, selection experiences, organizational conditions, stigma, and willingness to disclose psychological difficulties (Gulliver et al., 2012; Rice et al., 2016; Pascoe et al., 2022). These variables should be investigated directly rather than inferred from sex, residence, playing position, or competitive level.

The inclusion of clinical interviews, objective or behavioral measures of sleep, and information collected by multidisciplinary support teams could strengthen the interpretation of self-report findings. Further research should also examine whether psychological measures operate equivalently across relevant subgroups and evaluate the reliability, construct validity, incremental validity, sensitivity to change, and prospective utility of the composite psychological risk index. Such validation would be necessary before determining whether the index can meaningfully contribute to screening, referral, or intervention decisions.

## 5. Conclusions

Psychological profiles, sleep quality, and psychological risk indicators showed heterogeneous self-reported patterns across the teams included in this single elite football academy. Given the pooled cross-sectional design and the close interrelationship between sex, age, team, competitive level, and season, the observed differences should not be interpreted as independent effects of sex or competitive level, nor as evidence that particular groups have greater psychological vulnerability.

Exploratory differences were also observed according to competitive participation, playing position, living arrangements, years at the club, and body-composition status. However, these subgroup findings should be interpreted cautiously and cannot be considered independent associations without appropriate adjustment for relevant contextual and team-level factors.

The composite Psycholight index should be regarded as an exploratory descriptive operational summary of psychological and sleep-related indicators rather than as a validated decision-making tool. Further research is required to establish its psychometric properties, predictive validity, and practical utility before it can be recommended for systematic screening, referral, or intervention decisions.

## Figures and Tables

**Table 1:** Median Scores and Interquartile Ranges Across Team Categories.

	Motivation	Stress Control	Influence of Performance Evaluation	Maladaptive Perfectionism	Trait Anxiety	Anxiety Symptoms	Depression	Stress	Sleep Quality	Psychological Risk Index
***Men’s Teams***										
First team (M1)	**80 (65–85)** **^F1^**	82 (75–95)	**90 (80–95)** **^2/3, U16, F1^**	34 (25–37)	11 (1–30)	0 (0–2)	0 (0–4)	2 (0–6)	**5 (3–7)** **^M2/3, U19, U16^**	1 (1–2)
Second /Third teams (M2/3)	**80 (80–95)** ^F2^ *	**80 (70–90)** ^F2^ *	80 (75–90)	37 (30–41)	15 (5–40)	2 (0–4)	2 (0–4)	**4 (0–10)** **^M1^**	**4 (3–6)** **^U19, U16^**	1 (1–2)
U19	**85 (80–95)** **^M1, F3^**	**80 (65–90)** ^F3^ *	**85 (75–90)** **^F3^**	**38 (32–43)** **^M1^**	**25 (10–45)** **^M1^**	2 (0–4)	2 (0–4)	4 (0–8)	3 (2–5)	1 (1–2)
U16	**90 (80–95)** ^M1^ *^,2/3^	**80 (65–90)** ^U16F^ *	**85 (70–90)** **^U16F^**	**40 (34–48)** ^M1^ *^, M2/3^	20 (4–45)	**2 (0–4)** **^M1^**	**2 (0–6)** **^M1^**	**4 (0–10)** **^M1^**	3 (2–4)	1 (1–2)
***Women’s Teams***										
First team (F1)	70 (60–85)	65 (45–80)	80 (70–90)	35 (31–39)	**25 (11–50)** **^M1^**	**2 (0–4)** **^M1^**	**2 (0–6)** **^M1^**	**8 (2–12)** **^M1^**	**5 (3–8)** **^2, U16^**	**2 (1–3)** **^M1^**
Second team (F2)	70 (50–80)	65 (40–80)	75 (55–85)	37 (30–43)	30 (4–60)	2 (0–6)	2 (0–6)	6 (0–14)	4 (3–5)	**2 (1–4)** **^M2/3^**
Third team (F3)	80 (60–90)	55 (35–75)	75 (50–90)	**40 (35–46)** **^F1^**	40 (15–60)	2 (0–6)	**4 (2–8)** **^F1, F2, U19^**	**8 (4–14)** ^F2, U19^ *	**5 (3–6)** **^2, U16F, U19^**	**3 (2–4)** ^F1, F2, U19^ *
U16 (U16F)	85 (60–95)	40 (20–55)	55 (45–80)	**45 (38–49)** ^F1^ *	40 (15–77)	2 (0–6)	**4 (0–10)** **^F1, F2, U16^**	**8 (2–12)** **^U16^**	**4 (2–5)** **^U16^**	**3 (2–5)** ^F1, F2, U16^ *

Note. Labels within each cell indicate that the corresponding team showed significantly higher values than the teams identified in the label (*p* < 0.05). Asterisks indicate effect-size magnitude: no asterisk = small effect (0.10 ≤ r < 0.30); * = moderate effect (0.30 ≤ r < 0.50). Each team was compared with all teams of the same sex and with the opposite-sex team competing at the equivalent competitive level. Higher scores on motivation, stress control, and influence of performance evaluation indicate more favorable psychological profiles. Higher scores on maladaptive perfectionism, trait anxiety, anxiety symptoms, depression, stress, sleep quality, and the psychological risk index indicate less favorable psychological functioning or greater psychological distress. Anxiety symptoms were assessed using the anxiety subscale of the DASS-21. Light-blue shading distinguishes the men’s and women’s team sections. Bold formatting identify cells with statistically significant between-group comparisons.

**Table 2:** Distribution of Risk Categories Across Team Categories.

	Motivation	Stress Control	Influence of Performance Evaluation	Maladaptive Perfectionism	Trait Anxiety	Anxiety Symptoms	Depression	Stress	Sleep Quality	Psychological Risk Index
***Men’s Teams***										
First team (M1)	**52/30/18** ^M2/3^ *^, U19^ *^, U16^ **	81/11/8	85/11/4	85/15/0	80/15/5	91/1/8	89/7/4	95/2/3	**45/51/4** ^U19^ *^, U16^ *	86/8/6
Second /Third teams (M2/3)	76/23/1	80/17/3	80/15/5	72/25/3	68/24/8	92/4/4	91/5/4	92/4/4	**58/36/6** **^U19, U16^**	87/12/1
U19	79/13/8	72/18/10	82/12/6	**63/34/3** ^M1^ *	64/27/9	93/3/4	92/5/3	95/3/2	69/29/2	90/9/1
U16	85/13/2	72/18/10	76/13/11	**50/47/3** ^M1^ **^, M2/3^ *	64/23/13	**88/4/8** **^M2/3, U19^**	88/6/6	**91/5/4** **^U19^**	75/22/3	89/8/3
***Women’s Teams***										
First team (F1)	49/35/16	**45/27/28** ^M1^ **	80/14/6	84/14/2	63/24/13	86/5/9	84/7/9	89/6/5	**40/45/15** ^M2, U16^ *	78/16/6
Second team (F2)	**44/41/15** ^M2/3^ *	**38/31/31** ^M2/3^ **	64/21/15	69/28/3	51/33/16	**79/7/14** ^M2/3^ *	85/8/7	**82/10/8** ^F1, M2/3^ *	61/33/6	**64/26/10** ^F1, M2/3^ *
Third team (F3)	**54/33/13** ^U19^ *	**30/32/38** ^U19^ **	65/14/21	**52/46/2** ^F1^ **	44/44/12	**84/4/12** **^U19^**	**76/13/11** ^F1, F,2, U19^ *	**82/9/9** ^F1, U19^ *	**46/51/3** ^F2, F3, U19^ *	**55/36/9** ^F1^ *^, U19^ **
U16 (U16F)	68/16/16	**21/26/53** ^U16^ **	**42/32/26** ^F1^ **^, U16^ **	**37/47/16** ^F1^ **	37/26/37	83/5/12	**71/10/19** ^F1, F2, U16^ *	**80/10/10** **^F1, U16^**	**64/30/6** **^U16^**	**54/32/14** ^F1^ *^, U16^ **

Note. Labels within each cell indicate that players from the corresponding team had significantly higher odds of being classified in the medium- or high-risk categories than players from the teams identified in the label (*p* < 0.05). Asterisks indicate effect-size magnitude: no asterisk = small effect (1.5 ≤ OR < 2.5); * = moderate effect (2.5 ≤ OR < 4.0); ** = large effect (OR ≥ 4.0). Each team was compared with all other teams of the same sex and with the opposite-sex team competing at the equivalent competitive level. Light-blue shading distinguishes the men’s and women’s team sections. Bold formatting identify cells with statistically significant between-group comparisons.

**Table 3:** Distribution of risk categories (% of male players classified as low, medium, or high risk) according to playing position, years at the club, sum of six skinfolds, competitive participation, and living arrangements.

	Motivation	Stress Control	Influence of Performance Evaluation	Maladaptive Perfectionism	Trait Anxiety	Anxiety Symptoms	Depression	Stress	Sleep Quality	Psychological Risk Index
***Playing Position***										
Goalkeepers (GK)	65/32/3	81/16/3	74/16/10	58/42/0	68/19/13	95/3/2	95/2/3	95/2/3	70/26/4	**84/15/1** ^MF^ *
Center backs (CB)	66/22/12	73/24/3	80/15/5	63/34/3	59/27/14	88/5/7	89/5/6	93/4/3	66/33/1	**86/13/1** ^MF^ *
Midfielders (MF)	82/13/5	82/11/7	88/9/3	78/22/0	78/18/4	93/3/4	92/5/3	93/3/4	69/27/4	95/4/1
Wingers (W)	77/17/6	76/18/6	79/13/8	60/36/4	69/22/9	92/2/6	91/5/4	92/5/3	67/30/3	**90/8/2** **^MF^**
Forwards (FW)	86/11/3	77/14/9	77/18/5	66/30/4	61/32/7	90/4/6	**86/9/5** ^GK^ *^, MF^	93/3/4	65/33/2	**87/12/1** ^MF^ *
***Years at the Club***										
First-Year Players (1Y)	84/13/3	82/16/2	89/9/2	56/40/4	62/29/9	**84/6/10** ^≥2^ *	**86/7/7** **^≥2^**	**90/4/6** **^≥2^**	61/34/5	92/7/1
≥2 Years at the Club (≥2Y)	74/18/8	74/17/9	79/13/8	66/32/2	67/23/10	93/3/4	92/5/3	94/4/2	67/29/4	88/10/2
***Sum of Six Skinfolds (Team-Specific Tertiles)***										
Low (L)	72/18/10	66/26/8	80/13/7	70/30/0	67/20/13	91/2/7	91/5/4	93/5/2	68/27/5	90/9/1
Medium (M)	80/14/6	81/9/10	78/14/8	61/37/2	68/25/7	93/3/4	93/4/3	94/3/3	65/32/3	90/9/1
High (H)	70/26/4	79/16/5	84/10/6	72/25/3	71/21/8	90/4/6	**87/7/6** **^L, M^**	92/4/4	66/31/3	86/11/3
***Match Participation (Tertiles)***										
Low (L)	75/16/9	78/14/8	81/14/5	67/28/5	61/27/12	92/2/6	89/6/5	92/3/5	58/39/3	**84/15/1** **^H^**
Medium (M)	75/15/10	68/25/7	81/9/10	68/30/2	72/23/5	95/3/2	93/3/4	93/4/3	56/39/5	89/11/0
High (H)	65/29/6	87/9/4	84/15/1	76/24/0	73/22/5	92/3/5	92/5/3	94/3/3	64/31/5	92/7/1
***Living Arrangements***										
Family home (FH)	**77/18/5** **^OR^**	72/19/9	**75/17/8** **^OR^**	60/36/4	63/28/9	91/3/6	90/5/5	93/4/3	67/30/3	**87/11/2** **^ORes^**
Other residence (OR)	88/12/0	81/18/1	87/8/5	71/27/2	74/15/11	90/4/6	91/5/4	92/3/5	70/27/3	93/6/1

Note. Labels within each cell indicate that players in the corresponding group had significantly higher odds of being classified in the medium- or high-risk categories than players in the groups identified in the label (*p* < 0.05). Asterisks indicate effect-size magnitude: no asterisk = small effect (1.5 ≤ OR < 2.5); * = moderate effect (2.5 ≤ OR < 4.0). Light-peach shading distinguishes the sections corresponding to the different contextual variables. Bold formatting identify cells with statistically significant between-group comparisons. ORes = other residence.

**Table 4:** Distribution of risk categories (% of female players classified as low, medium, or high risk) according to playing position, years at the club, sum of six skinfolds, competitive participation, and living arrangements.

	Motivation	Stress Control	Influence of Performance Evaluation	Maladaptive Perfectionism	Trait Anxiety	Anxiety Symptoms	Depression	Stress	Sleep Quality	Psychological Risk Index
***Playing Position***										
Goalkeepers (GK)	54/31/15	31/31/38	69/23/8	62/38/0	54/38/8	**76/8/16** **^MF, W^**	84/9/7	84/9/7	70/28/2	61/33/6
Center backs (CB)	44/32/24	35/29/36	74/21/5	71/29/0	53/41/6	84/5/11	80/9/11	85/10/5	**49/43/8** **^GK^**	71/27/2
Midfielders (MF)	49/33/18	37/30/33	70/21/9	67/30/3	47/42/11	87/4/9	78/10/12	**82/12/6** **^W^**	**48/43/9** **^GK^**	64/29/7
Wingers (W)	57/36/7	43/23/34	68/9/23	64/32/4	57/23/20	86/5/9	82/9/9	89/4/7	**56/37/7** **^GK^**	68/23/9
Forwards (FW)	62/29/9	29/29/42	43/33/24	52/38/10	33/38/29	**75/8/17** **^CB, MF, W^**	79/10/11	**75/9/16** ^CB, W^ *	**50/42/8** **^GK^**	**55/28/17** **^CB, W^**
***Years at the Club***										
First-Year Players (1Y)	56/33/11	56/22/22	78/17/5	67/33/0	61/33/6	87/5/8	85/10/5	88/4/8	65/27/8	83/12/5
≥2 Years at the Club (≥2Y)	53/31/16	32/29/39	61/20/19	63/33/4	46/36/18	83/5/12	79/10/11	84/9/7	**51/43/6** **^1Y^**	**62/29/9** ^1Y^ *
***Sum of Six Skinfolds (Team-Specific Tertiles)***										
Low (L)	57/29/14	33/31/36	63/20/17	65/35/0	47/33/20	85/6/9	85/7/8	89/6/5	57/33/10	66/27/7
Medium (M)	41/48/11	30/30/40	63/20/17	57/41/2	43/39/18	82/5/13	**79/9/12** **^L^**	85/8/7	51/42/7	68/24/8
High (H)	52/29/19	41/25/34	68/18/14	68/23/9	54/36/10	81/5/14	**75/12/13** **^L^**	**76/13/11** ^L^ *^, M^	**46/48/6** **^L^**	**59/30/11** **^L^**
***Match Participation (Tertiles)***										
Low (L)	56/28/16	34/24/42	62/16/22	58/36/6	40/36/24	**79/5/16** **^M, H^**	**75/10/15** **^H^**	**80/9/11** **^M^**	**49/42/9** **^H^**	**57/24/19** **^H^**
Medium (M)	36/43/21	32/30/38	**50/34/16** ^A^ *	61/39/0	50/34/16	85/4/11	80/9/11	88/6/6	**43/49/8** **^H^**	**55/40/5** ^H^ *
High (H)	60/28/12	40/30/30	77/9/14	72/23/5	63/28/9	85/7/8	84/10/6	83/10/7	62/34/4	77/20/3
***Living Arrangements***										
Family home (FH)	54/35/11	29/36/35	60/19/21	54/41/5	47/36/17	**81/5/14** **^ORes^**	**77/11/12** **^ORes^**	**80/10/10** **^ORes^**	56/39/5	**58/31/11** **^ORes^**
Other residence (OR)	42/38/20	46/8/46	79/12/9	71/29/0	42/46/12	88/5/7	85/7/8	87/8/5	52/42/6	68/27/5

Note. Labels within each cell indicate that players in the corresponding group had significantly higher odds of being classified in the medium- or high-risk categories than players in the groups identified in the label (*p* < 0.05). Asterisks indicate effect-size magnitude: no asterisk = small effect (1.5 ≤ OR < 2.5); * = moderate effect (2.5 ≤ OR < 4.0). Light-peach shading distinguishes the sections corresponding to the different contextual variables. Bold formatting identify cells with statistically significant between-group comparisons. ORes = other residence.

## Data Availability

The data are not publicly available because of the sensitive and confidential nature of the psychological information and the inclusion of underage players. Requests for access may be considered subject to authorization by Athletic Club and compliance with the applicable data-protection requirements.

## Abbreviations

CPRD	Psychological Characteristics Related to Sport Performance Questionnaire
MIPS	Multidimensional Inventory of Perfectionism in Sport
STAI	State–Trait Anxiety Inventory
DASS-21	Depression Anxiety Stress Scales–21
PSQI	Pittsburgh Sleep Quality Index
OR	Odds ratio
U16	Under-16
U19	Under-19
GK	Goalkeeper
CB	Center back
MF	Midfielder
W	Winger
FW	Forward
FH	Family home
ORes	Other residence
L	Low
M	Medium
H	High
